# Red Fox as Sentinel for *Blastomyces dermatitidis*, Ontario, Canada

**DOI:** 10.3201/eid2207.151789

**Published:** 2016-07

**Authors:** Nicole M. Nemeth, G. Douglas Campbell, Paul T. Oesterle, Lenny Shirose, Beverly McEwen, Claire M. Jardine

**Affiliations:** Canadian Wildlife Health Cooperative, Guelph (N.M. Nemeth, G.D. Campbell, P.T. Oesterle, L. Shirose, C.M. Jardine);; University of Guelph, Guelph, Ontario, Canada (N.M. Nemeth, P.T. Oesterle, B. McEwen, C.M. Jardine)

**Keywords:** Blastomyces dermatitidis, blastomycosis, fungal disease, canid, dog, fungi, red fox, Vulpes vulpes, sentinel, Canada

## Abstract

*Blastomyces dermatitidis*, a fungus that can cause fatal infection in humans and other mammals, is not readily recoverable from soil, its environmental reservoir. Because of the red fox’s widespread distribution, susceptibility to *B. dermatitidis*, close association with soil, and well-defined home ranges, this animal has potential utility as a sentinel for this fungus.

*Blastomyces dermatitidis* (family Ajellomycetaceae) is a fungal pathogen that causes blastomycosis, a life-threatening disease in humans, canids, and other mammals (*1*,*2*). Infection usually occurs through inhalation of conidia released from an environmental reservoir (soil) (*3*). In North America, high incidences of infection are reported in humans and dogs from around the Great Lakes (*4*). Recently, increased numbers of human blastomycosis cases have been detected in the provinces of Quebec, Saskatchewan, Manitoba, and Ontario, Canada (*2*,*5*–*7*). Despite this increased detection, human blastomycosis is probably underdiagnosed (*2*,*8*).

The geographic range of *B. dermatitidis* is based on reported clinical human cases and is therefore not clearly defined (*4*). *B. dermatitidis* is not readily recoverable or uniformly distributed within the environment, and identification has been problematic (*4*,*9*). Therefore, identifying high-risk areas for exposure has been difficult, yet this this information is crucial to minimize the number of infections.

We evaluated the utility of wild and domestic canids as potential sentinels of *B. dermatitidis* in the environment. We retrospectively reviewed blastomycosis case data for wildlife and companion animals in Ontario, which contains areas where blastomycosis is endemic and areas of likely emergence (*2*,*3*,*5*,*8*). Once a candidate sentinel species is identified, a targeted surveillance system can be developed to identify high-risk areas and assess risk factors associated with disease.

## The Study

We analyzed blastomycosis cases diagnosed at the Animal Health Laboratory, University of Guelph (Guelph, Ontario), during 1998–2014; at 2 private diagnostic services (Guelph) during 1996–2006; and at the Canadian Wildlife Health Cooperative (Ontario regional center) during 1991–2014. Case data included date of sample collection, species, and location of carcass (wildlife) or veterinary clinic or diagnostic laboratory (companion animals). Personal privacy legislation in Canada prevented use of home addresses for companion animals. We compared animal blastomycosis data with those for 309 published human cases in Ontario during 1994–2003 (*2*).

Diagnoses in companion animals were made using impression smears, cytology, histopathology, serology (agar gel immunodiffusion test), and antigen detection (sandwich enzyme immunoassay). Diagnoses in wildlife were made postmortem by gross pathology and histopathology.

Blastomycosis was diagnosed in 250 companion animals (222 dogs [88.8%], 27 cats [10.8%], 1 ferret [0.4%]) and in 14 wild canids (11 red foxes [*Vulpes vulpes*; 78.6%], 3 gray wolves [*Canis lupus*; 21.4%]). Diagnoses in wild canids represent 7.4% of 149 red foxes and 1.6% of 185 wolves submitted to the Canadian Wildlife Health Cooperative (Ontario regional center) during the same period. Lungs of wild canids with blastomycosis consistently had nodules of inflammatory cells and *B. dermatitidis* yeasts. Less commonly, lymph nodes and skin were also affected. In red foxes found dead, *B. dermatitidis* was associated with severe, multifocal to coalescing, granulomatous pneumonia, whereas trapper-killed red foxes had small numbers of well-circumscribed pulmonary lesions.

Most infected companion animals were from central regions of Ontario, as previously defined (*2*); however, all regions were represented (Figure). All infected wild canids were in the north region, where most human blastomycosis cases (61%; 188/309) originated during 1994–2003 (*2*). An additional study traced 74% (20/27) of human cases to north and east of Lake Superior (*3*).

**Figure:**
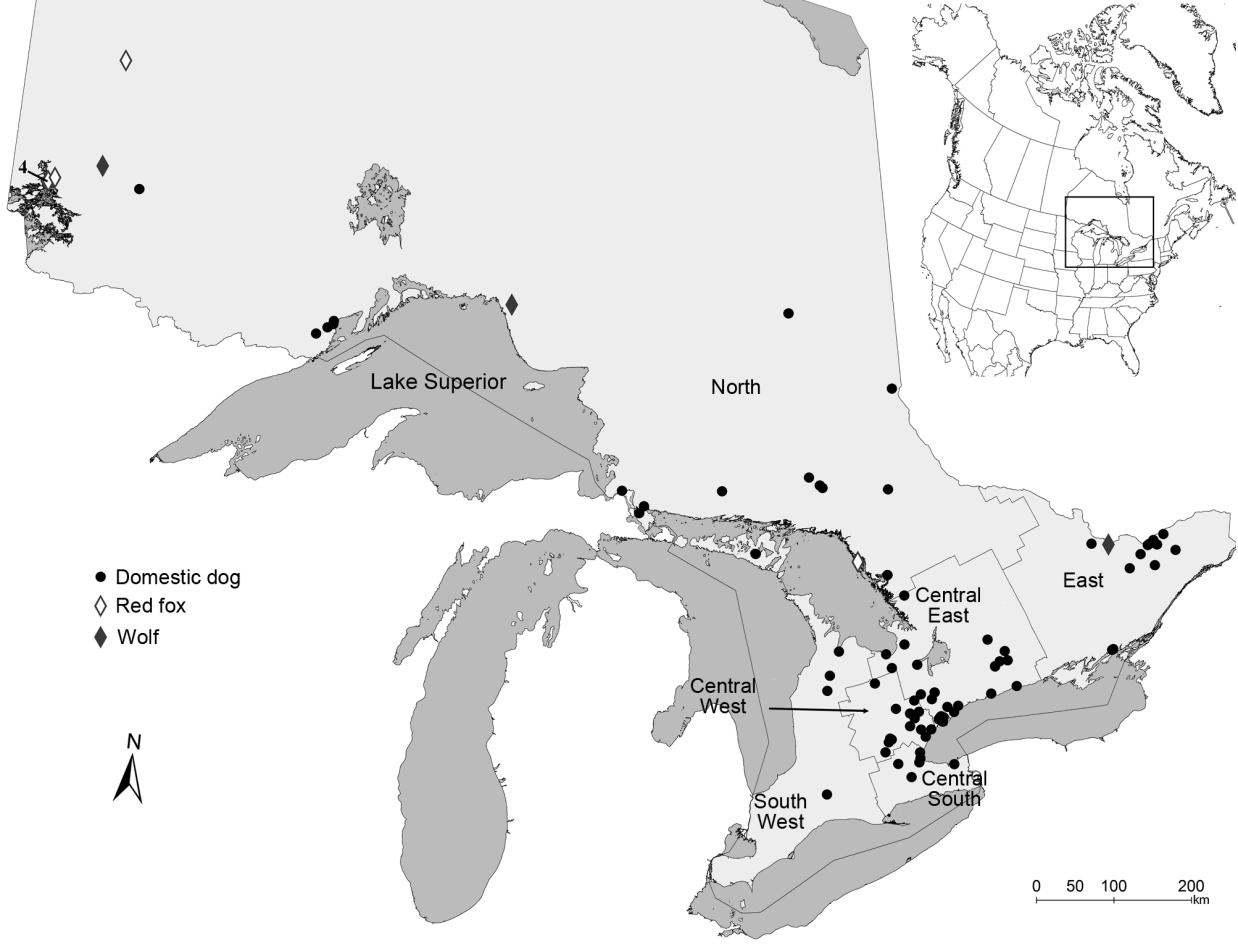
Locations of wild and domestic canids infected with *Blastomyces dermatitidis* during 1996–2014, Ontario, Canada. Inset map shows the location of Ontario in Canada. Health regions within the province consist of grouped public health units as defined by the Ontario Ministry of Public Health and are named according to Morris et al. (*2*). Dark gray shading indicates lakes; the Great Lakes are shown in the lower part of the figure.

Dogs were most commonly diagnosed with blastomycosis (64.4%) during July–December; the fewest (14%) were diagnosed during January–March. Most wildlife with blastomycosis (85.7%, 10/11 red foxes and 2/3 wolves) were diagnosed during November–March; the remaining 2 animals were diagnosed in September and April. Most human cases (59%, 181/309) were diagnosed during October–March (*2*).

*B. dermatitidis* infections in humans and other mammals are opportunistic and associated with contact with aerosolized conidia. Habitat sharing among humans, wildlife, and domestic animals is increasingly common (*10*) and provides communal opportunities for *B. dermatitidis* exposure. Wildlife with limited and relatively well-defined home ranges (e.g., the red fox) offer a unique opportunity to assess the distribution and, therefore, the potential risks of exposure to pathogens with an environmental reservoir.

The red fox is the most widely distributed carnivore worldwide and is common throughout much of North America, including regions with endemic as well as emerging *B. dermatitidis* (*11*). This species is highly adaptive and coexists with humans in various habitats, including recreational, residential, commercial, industrial, and urban open areas (*10*). Furthermore, the home range of the red fox is compact, nonoverlapping, and relatively well-defined with year-round occupancy, unlike the range of other wild canids (*10*,*12*), humans, and dogs, which can have varied and long-distance movements. The close proximity of red foxes to the ground, along with their digging, denning, and foraging behaviors, probably increases the likelihood of their close and continuous exposure to *B. dermatitidis* in the soil. In addition, soil in red fox dens is often sandy and acidic, a condition that, along with moisture, decaying vegetation, and animal feces, is conducive to *B. dermatitidis* growth (*1*,*3*,*11*).

Based on the co-occurrence of clinical disease in dogs and their owners, canine blastomycosis cases are a potential epidemiologic marker for the risk for human disease (*9*). However, in our study, pinpointing environmental exposure sources for dogs was impossible due to undisclosed travel, privacy legislation, and location variation between exposure sites and clinics. The time lag between exposure and disease onset can vary by months (*13*), further hindering accurate identification of exposure sources. In our study, dog blastomycosis cases mapped to Guelph are overrepresented because this southwestern Ontario city has a large veterinary hospital and diagnostic laboratory.

Although relatively few wild, compared with domestic, canids were diagnosed with blastomycosis in our study, the utility of an abundant and widespread wild canid such as the red fox as a sentinel for the risk for *B. dermatitidis* infection in humans should be further explored. In our study, *B. dermatitidis* was readily detected by gross pathology and histopathology in the lungs of red foxes, in which pulmonary lesions ranged from severe, diffuse pneumonia in foxes found dead to focal and well-circumscribed lesions in trapper-killed foxes. These findings suggest that *B. dermatitidis*–associated lesions in red foxes would be easily identifiable, regardless of time of year and disease manifestation. Future sampling should target foxes in northern Ontario, where risk for human infections is highest, the annual incidence is increasing, and diagnostic testing is less available (*2*,*8*,*14*). Furthermore, in remote northern regions, defining the range and prevalence of blastomycosis could have positive public health effects on healthcare workers and indigenous human populations.

Limitations for using wildlife for passive disease surveillance include difficulty in finding and recovering carcasses, leading to inconsistent sampling efforts. To circumvent the limitations, we suggest using carcasses of licensed fur trapper–killed red foxes for testing in targeted areas and seasons (i.e., fall to early spring). Red fox trapping is permitted year-round in southern Ontario (*2*) and from September to late February in the remainder of the province (*15*). Temporal detections of blastomycosis in humans in Ontario more closely followed detections in red foxes than in dogs.

## Conclusions

Documenting the prevalence, distribution, seasonality, and disease manifestations of blastomycosis in red foxes in southern Ontario could help elucidate the epidemiology of this regionally emerging disease, delineate geospatial differences in exposure risks, and explore the utility of this wild canid as a sentinel for the risk to public health. Multidisciplinary research such as this provides opportunities for the development of partnerships among public health and medical researchers, physicians, veterinarians, biologists, epidemiologists, natural resource managers, and hunter and trapper federations with the common goal of reducing disease risks.
